# Proliferation capability of natural killer cells upon cytokines stimulation correlated negatively with serum lactate dehydrogenase level in coronary artery disease patients

**DOI:** 10.3389/fimmu.2024.1436747

**Published:** 2024-09-02

**Authors:** Xuemin Guo, Ting Xiao, Li Lin, Qianqian Gao, Bifa Lai, Xianhui Liu, Zhixiong Zhong

**Affiliations:** ^1^ Institute of Basic Medical Sciences, Meizhou People’s Hospital, Meizhou, China; ^2^ Guangdong Engineering Technological Research Center for Clinical Molecular Diagnosis and Antibody Drugs, Meizhou, China; ^3^ Department of Neuroscience, City University of Hong Kong, Kowloon, Hong Kong, Hong Kong SAR, China

**Keywords:** natural killer (NK) cell, coronary artery disease, stimulatory factor, proliferative response, lactate dehydrogenase

## Abstract

**Background:**

Natural killer (NK) cells are proposed to participate in coronary artery disease (CAD) development. However, little is known about how CAD patients’ NK cells respond to different stimulatory factors in terms of proliferation capability.

**Methods and results:**

Twenty-nine CAD patients’ peripheral blood NK cells were isolated and individually treated with IL-2, IL-12, IL-15, IL-18, IL-21, cortisone acetate, hydrocortisone, or ascorbic acid for 36 hours, followed by cell cycle analysis using flow cytometry. The ratio of S and G2/M phase cell number to total cell number was defined as a proliferation index (PrI) and used for proliferative capability indication. The results showed that these eight factors resulted in different life cycle changes in the 29 NK cell samples. Remarkably, 28 out of 29 NK cell samples showed an obvious increase in PrI upon ascorbic acid treatment. The serum lactate dehydrogenase (LDH) level of the 29 CAD patients was measured. The results showed a negative correlation between serum LDH level and the CAD patients’ NK cell PrI upon stimulation of interleukins, but not the non-interleukin stimulators. Consistently, a retrospective analysis of 46 CAD patients and 32 healthy donors showed that the circulating NK cell number negatively correlated with the serum LDH level in CAD patients. Unexpectedly, addition of LDH to NK cells significantly enhanced the production of IFN-γ, IL-10 and TNF-α, suggesting a strong regulatory role on NK cell’s function.

**Conclusion:**

Ascorbic acid could promote the proliferation of the CAD patients’ NK cells; LDH serum level may function as an indicator for NK cell proliferation capability and an immune-regulatory factor.

## Introduction

Natural killer (NK) cells are an important part of the innate immune system and comprise the third largest component of the lymphocyte population, after T cells and B cells (1). Except for directly killing target cell through the release of perforin- and granzyme-containing cytotoxic granules, NK cells also act as regulatory cells in the immune system by producing a panel of various cytokines and chemokines, such as IFN-γ, TNF-α, GM-CSF, IL-5, IL-10, and IL-13 (2, 3).

NK cells not only play important anti-tumor and anti-virus roles, but are also involved in chronic inflammatory conditions, such as atherosclerosis (AS) (4). AS is one important pathological basis of coronary artery diseases (CAD) (5, 6). Many types of immune cells are involved in the AS development and CAD progression. In which, macrophage and T cells are well studied, but not NK cells. Previous studies have reported a reduction in NK cells and a concomitant loss of NK cell activity in patients with CAD (7, 8). NK cells mainly modulate the inflammatory response in CAD patients by activating inflammation and affecting cardiac remodeling by significantly increasing the expression of TNF-α and IL-10 (9, 10). A twelve-month follow-up to 43 CAD patients showed that persistent NK cell deficiency was associated with low-grade cardiac inflammation (8). However, the influencing factors and underlying mechanisms remain elusive.

Impaired NK cell activity and low NK cell number have been observed in patients with cancer (11, 12). In recent years, there have been many studies on the use of NK cells in cancer immunotherapy (2, 13). The antitumor activity of NK cells could be greatly increased through *ex vivo* activation, expansion and genetic modification. Some of these methods have been translated into clinical-grade platforms and shown promising results in the clinical trials of NK cell infusions in patients with hematological malignancies or solid tumors (2). Interleukins, such as IL-2, IL-12, IL-15, IL-18, and IL-21, glucocorticoids, such as cortisone acetate and hydrocortisone, and ascorbic acid can promote the *ex vivo* proliferation of NK cells in healthy people or cancer patients (14–16). IL-12, IL-15 and IL-18 are powerful inducers of IFN-γ production when used in combination (17, 18). Given the potential role of NK cells in cancer immunotherapy, more efficient *in vitro* methods for expansion of clinical grade NK cells need to be developed.

Interleukin, ascorbic acid and glucocorticoid have been widely studied in cancer patients, which showed different stimulation on NK cell proliferation and activation (4, 19, 20). In contrast, little is known about how NK cells in CAD patients respond to interleukins, glucocorticoids, and ascorbic acid in proliferative capability. Given that NK cells are deeply involved in atherosclerosis development and CAD progression by showing a reduction in circulating cell number and cell activity (5–8), it’s important to explore the effects of these stimulatory factors on NK cell proliferation and function in CAD patients, which will facilitate the clinical significance of NK cells in CAD management. Here we investigated the effects of eight different stimulatory factors, including IL-2, IL-12, IL-15, IL-18, IL-21, cortisone acetate (CA), hydrocortisone (HC), and ascorbic acid (AA), on the *ex vivo* proliferation of 29 CAD patients’ NK cells, and correlated the results with the serum level of myocardial enzymes, including creatine kinase (CK), creatine kinase isoenzyme (CK-MB), and lactate dehydrogenase (LDH). The influence of LDH on NK cells life cycle and the production of IFN-γ, IL-10 and TNF-α was also investigated. Meanwhile, the relationship between serum LDH level and the number of circulating NK cells was retrospectively analyzed in CAD patients.

## Materials and methods

### Patients and data collection

CAD patients and healthy donors (HDs) with no evidence of any disease and infection were recruited at Meizhou People’s Hospital from May 2020 to December 2022. The hospitalized patients with newly diagnosed CAD were included, including myocardial infarction, heart failure, coronary atherosclerotic heart disease, *etc.* Our research excluded the patients with current infection, long-term anti-inflammatory medication usage, malignancy, severe gastrointestinal diseases and renal insufficiency, as well as the individuals with allergy or rheumatoid diseases. A total of 37 CAD patients and 22 healthy donors (HDs) with no evidence of any disease and infection were eventually enrolled throughout this research. Among them, 29 CAD patients and 12 HDs entered into the study of NK cell proliferation upon stimuli; the residual 8 CAD patients and 10 HDs entered into the study of NK cell secreting cytokines upon stimuli. Before inclusion in this study, informed consent was signed by each CAD patient and HDs, and the research protocol were approved by the ethical committee of Meizhou People’s Hospital. The clinical information of the enrolled CAD patients and HDs were collected, including age, gender, blood pressure, serum lipid levels, Liver function indicators, cardiac marker enzymes, and lymphocyte numbers. Meanwhile, the clinical data of 46 CAD patients and 32 HDs subjected to lymphocytes analysis with FCM were collected and used for retrospective analysis.

### Preparation of peripheral blood NK cells

Peripheral blood samples (10 mL) were obtained from the enrolled patients and HDs. The blood sample collection procedure conformed to the informed consent guidelines. Peripheral blood mononuclear cells (PBMCs) were prepared according to Choi et al. (13). After wash three times with phosphate-buffered saline (PBS), pH 7.2, the obtained PBMCs were re-suspended with 40 μL MACS solution, i.e. PBS, pH 7.2, supplemented with 2 mM EDTA (Sigma-Aldrich, St Louis, MO, USA) and 0.5% bovine serum albumin (BSA; Sigma-Aldrich). NK cells were then prepared by using a NK cell negative selection kit (Miltenyi Biotec, Bergisch Gladbach, Germany) according to the manufacturer’s instructions. Briefly, 10 μL NK cell biotin-antibody cocktail was added to the 40 μL PBMCs suspension and incubated at 4°C for 5 min, followed by addition of 30 μL of MACS solution and 20 μL of NK cell MicroBead cocktail. After incubation at 4°C for 10 min, the NK cells were enriched through a magnetic separator, followed by purity detection using flow cytometry (BD FACS CantoII). The purity of the NK cells routinely exceeded 95%, with one flow cytometry plot shown in **Supplementary Figure S1** as a representative.

### Cell cultures

Prepared NK cells were suspended in RPMI-1640 medium (Gibco, Australia) supplemented with 10% fetal bovine serum (FBS; Gibco, Australia). After counting, the NK cells were divided into nine groups and seeded onto 24-well plates at 5×10^5^ cells/mL. Eight out of the nine groups were co-cultured with 10 ng/L of IL-2, 10 ng/mL of IL-12, 20 ng/mL of IL-15, 200 ng/mL of IL-18, 100 ng/mL of IL-21, 1 μmol/L of CA, 0.5 μmol/L of HC, and 100 mg/L of AA. All stimulatory factors were purchased from Meilun Biotech (Dalian, China). The residual group without any treatment was used as a control. NK cells from these nine groups were then incubated for 36 h at 37°C in a 5% CO_2_ incubator. The commonly used dose of the stimulatory factors and the treatment time were selected based on multiple literatures (13, 19, 21–23).

### Cell cycle assay through flow cytometry

The NK cells in each well with or without stimulatory factor treatment were collected individually, washed twice with PBS, and then suspended in a 50 μL fixative solution provided in the IntraSure kit (BD Biosciences, Franklin Lakes, USA) according to the manufacture’s instruction. After incubation at 4°C for 1 h, the cells were washed with PBS, and then treated with 10 μL of membrane breaker for 15 min. Following fixation cells were treated with 100 μg/mL RNase A (ThermoFisher Scientific, United States), the cells were incubated with 50 μg/mL propidium iodide (PI; Invitrogen, Eugene, OR, USA) in the dark for 15 min after wash with PBS. A minimum of 10,000 cells were then immediately analyzed using FACSCanto flow cytometer (BD Biosciences). The results were analyzed by FlowJo7.6. The cell cycle four phases (including G1, S, G2, and M) of the treated NK cells was recognized as described previously (24). Same performance was done to the untreated NK cells as control. The ratio of S and G2/M phase cell number sum to total cell number was defined as a proliferation index (PrI) and used for proliferative capability indication.

### Detection of myocardial enzymes

About 2 mL peripheral blood was collected from the 29 CAD patients and 12 HDs as mentioned above, and serum was then isolated. Beckman AU5800 was used to detect the serum level of CK, CK-MB, LDH, α-hydroxybutyrate dehydrogenase (α-HBDH). The reagents were provided by MedicalSystem Biotec (Jiangsu, China). Abbott Alinity and its matching reagents were used to detect brain natriuretic peptide (BNP) (Pennsylvania, USA) and cardiac troponin I (cTnI) (Longford, Irland).

### Circulating NK cell counting

The clinical data of 46 CAD patients and 32 HDs subjected to lymphocytes analysis with FCM were analyzed retrospectively, including age, gender, blood pressure, serum lipid levels, Liver function indicators, cardiac marker enzymes, and lymphocyte numbers. The percentages and absolute numbers of T cells, B cells and NK cells in peripheral blood were determined by using TruCOUNT tubes and BD Multitest 6-color TBNK Reagent Kit with FACSCanto flow cytometer using FACSCanto clinical software (BD Biosciences, USA).

### Measurement of secreted cytokines via ELISA assay

As described above, a total of 8 CAD patients and 10 HDs were enrolled and their clinical information was collected. The research protocol was approved by the ethical committee of Meizhou People’s Hospital. The peripheral blood NK cells were purified as described above and then re-suspended in RPMI-1640 medium to reach a final concentration at 8×10^5^/mL. The cell suspension was seeded onto a 24-well plate with 500 μL each well, and then co-cultured with each of eight stimulatory factors as well as LDH (1000 U/L; Sigma-Aldrich) at 37°C for 80 h. After centrifugation at 4°C for 10 min, the cell-free supernatants were harvested and the level of IFN-γ, IL-10 and TNF-α was subsequently measured by using the corresponding ELISA Kits (OriGene Tech, Rockville, MD, USA) according to the manufacturer’s instructions. O.D. values were recorded at 450 nm. The standard curve was created according to the O.D. values of the standards. The quantities of these cytokine secreted by NK cells were calculated from the standard curves.

### Statistical analysis

IBM SPSS Statistics 21.0 (IBM, Armonk, NY, USA) was used for data analysis. Data were presented as mean ± SD, and the results were analyzed using Student’s *t* test. Spearman’s test was used for correlation analyses. Statistical significance was accepted when p < 0.05.

## Results

### The effects of eight stimulatory factors on NK cell cycle in CAD patients

**Figure 1:**
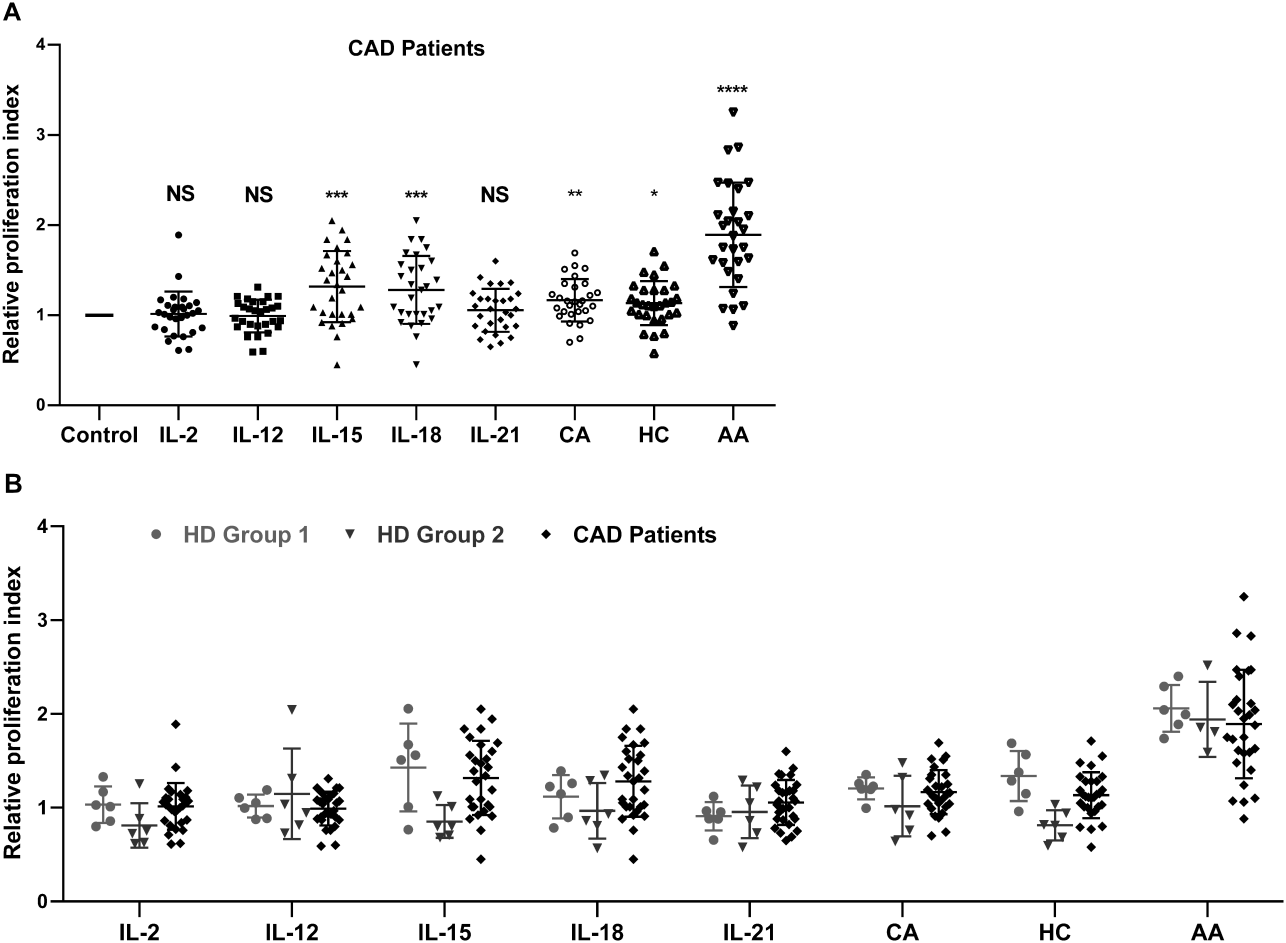
The effect of eight stimulatory factors on the proliferation of NK cells *in vitro*. The relative proliferation index (R-PrI) of peripheral NK cells from 29 CAD patients **(A)** and 12 healthy controls were used in comparison with the CAD patients **(B)** after treatment with stimulatory factors was shown. Each scatter point indicates the ratio of PrI of NK cells after respective stimulation by IL-2, IL-12, IL-15, IL-18, IL-21, cortisone acetate (CA), hydrocortisone (HC), and ascorbic acid (AA) to PrI of NK cells without stimulation. The later one was used as control with the PrI set as 1. Data were presented as mean ± SD; comparison between the control group and other experimental groups, *P < 0.05, **P < 0.01, ***P < 0.001, ****P < 0.0001, NS means no statistical significance.

**Figure 2:**
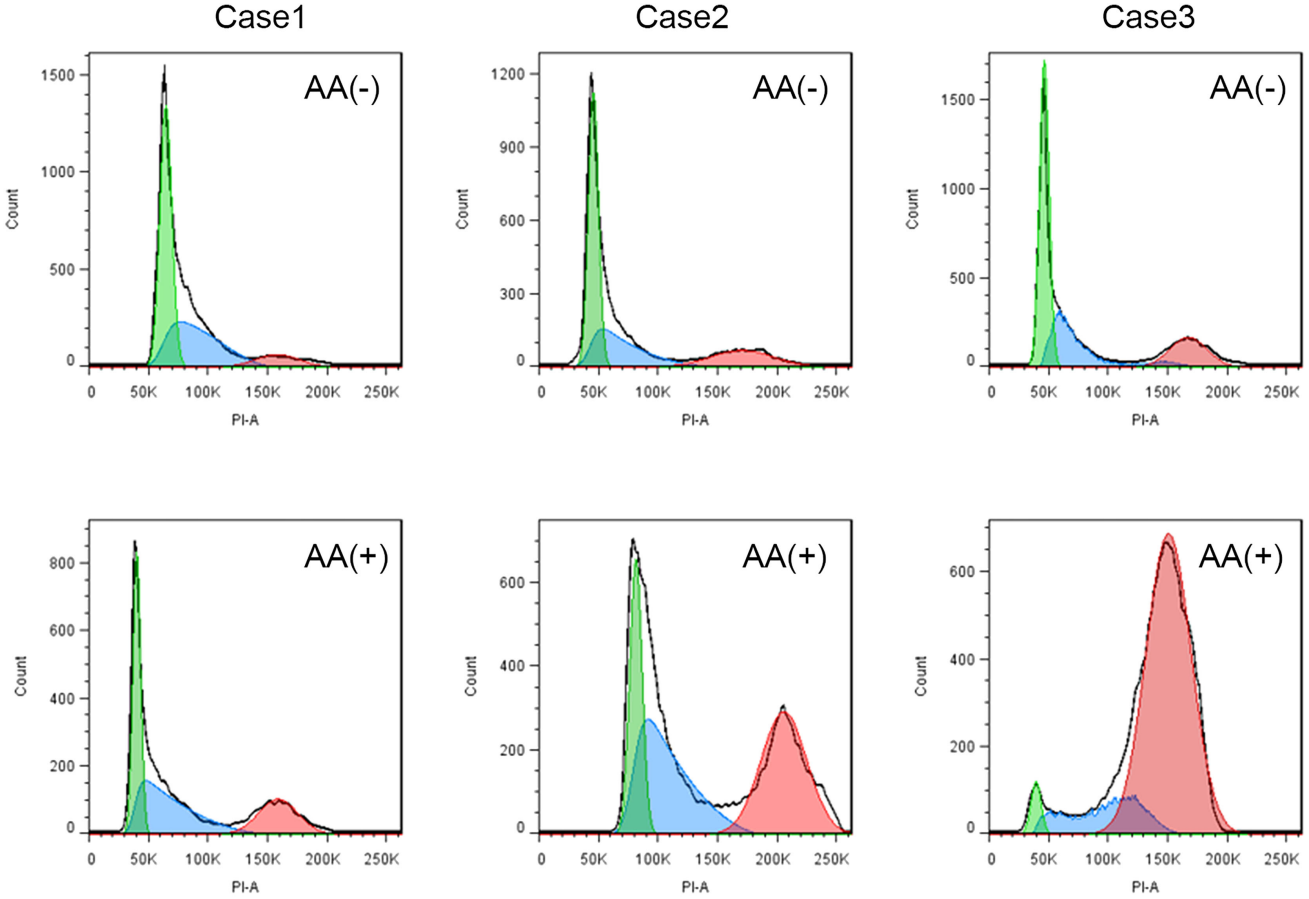
The representative results of low, medium, and high PrI for NK cells with ascorbic acid treatment. AA (-) is a schematic flow cytometry plot showing the cell cycle of CAD patient NK cells without ascorbic acid treatment, and AA (+) is a schematic diagram of the cell cycle of CAD patient NK cells stimulated by ascorbic acid.

The effects of IL-2, IL-12, IL-15, IL-18, IL-21, CA, HC, and AA on the proliferation of peripheral blood NK cells were analyzed. The clinical characteristics of the enrolled 29 CAD patients and 12 HDs. were shown in **Table 1**. The HDs were divided into two groups; group2 matched the CAD patients in age and group 1 was younger. The NK cells samples from these subjects were individually stimulated with each of the eight stimulatory factors or without. After which, flow cytometry was used to analyze the effects of these factors on the cell cycle of NK cells. The ratio of the stimulated NK cells PrI to the unstimulated NK cells PrI was defined as relative proliferation index (R-PrI). The PrI of the NK cells without stimulatory factor treatment was set as 1. As shown in **Figure 1A** and **Supplementary Table S1**, the proliferative promotion of IL-15, IL-18, CA, HC, and AA on the 29 CAD patients’ samples was significant. NK cells derived from either HDs or CAD patients showed similar tendency in proliferative responses to the eight stimulatory factors (**Figure 1B**). Upon stimulation of five interleukins and two glucocorticoids, no matter HDs or CAD patients, some NK cell samples had R-PrI >1, while others had R-PrI <1, and the increase or decrease of each group was inconsistent (**Figure 1B**), suggesting a heterogeneous response of NK cells to the stimuli in genomic DNA replication. In contrast, AA had the most significant effect on promoting NK cells entry into S/G2 phases in both HDs and CAD patients. NK cells from all HDs and 28 out of 29 CAD patients had R-PrI >1 after treatment with AA, and the increase was significant compared to other stimulators’ treatment. **Figure 2** shows the representative results of low, medium, and high PrI of NK cells after AA treatment. When PrI was high, genomic DNA replication was active, and essentially more cells were in the S and G2/M phases. These results indicated that AA could extensively and significantly stimulate the proliferation of peripheral blood NK cells in either HDs or CAD patients, while interleukins and glucocorticoids just showed weaker stimulation effects on NK cells.

**Table 1:** Characteristics of CAD patients and healthy donors enrolled for NK cell *in vitro* proliferation assay.

	CAD patients	Healthy donors	
		Group 1	Group 2
Age (years)	72 (9.42)	55.3 (6.11)	73.67 (4.13)
Male/female (n)	18/11	3/3	4/2
Blood pressure			
Syst (mmHg)	138.97 (21.56)	128.4 (11.6)	132 (15.25)
Diast (mmHg)	81.76 (13.12)	80.5 (6.4)	79.17 (9.62)
Laboratory variables			
TC (mmol/L)	4.62 (1.26)	4.86 (1.01)	4.32 (0.85)
LDL-C (mmol/L)	2.67 (0.99)	2.35 (0.57)	2.45 (0.72)
HDL-C (mmol/L)	1.28 (0.29)	1.49 (0.43)	1.52 (0.38)
Apo-A1 (g/L)	1.07 (0.22)	1.16 (0.55)	1.31 (0.32)
Apo-B (g/L)	0.89 (0.29)	0.82 (0.13)	0.71 (0.16)
TG (mmol/L)	1.40 (0.79)	1.24 (0.36)	1.18 (0.49)
Hcy (µmol/L)	20.90 (9.42)	9.83 (4.49)	14.41 (2.57)
Glu (mmol/L)	6.40 (2.54)	4.94 (0.93)	5.17 (0.41)
Leukocyte subsets			
WBC (×10^9/^L)	6.43 (2.88)	6.71 (0.31)	5.85 (0.98)
NEUT (×10^9/^L)	4.24 (2.86)	4.08 (0.22)	3.5 (0.56)
LYMPH (×10^9/^L)	1.57 (0.70)	1.66 (0.38)	1.91 (0.51)
Mon (×10^9/^L)	0.39 (0.29)	0.41 (0.12)	0.42 (0.15)
RBC (×10^12/^L)	4.14(0.73)	4.24 (0.56)	4.52 (0.47)
Hb (g/L)	122.24(21.63)	116 (15.81)	144.17 (15.46)
Liver function indicators			
ALT (U/L)	27.44 (17.07)	22.80 (12.37)	24.50 (11.67)
AST (U/L)	34.44 (33.11)	24.18 (11.03)	26.33 (4.68)
ALP (U/L)	73.41 (23.62)	79.01 (12.68)	68.17 (12.58)
GGT (U/L)	42.56 (20.95)	26.43 (10.52)	23.00 (13.48)
Cardiac marker			
CK (U/L)	143.55 (132.96)	110.66 (42.83).	104.00 (49.51)
CK-MB (U/L)	17.17 (9.73)	15.73 (5.92)	16.2 (4.50)
LDH (U/L)	263.27 (152.82)	192.5 (50.67)	184.40 (35.32)
α-HBDH (U/L)	232.73 (174.70)	165.31 (70.9)	150.60 (34.89)
BNP (pg/ml)	518.04 (625.69)	17.58 (9.74)	29.28 (25.35)
cTnI (ng/ml)	0.09 (0.15)	0.0057 (0.005)	0.0086 (0.0052)

Values are given as mean (SD); Syst, systolic pressure; Diast, diastolic pressure; TC, total cholesterol;

LDL-C, low density lipoprotein cholesterol; HDL-C, high density lipoprotein cholesterol; Apo, apoprotein;

TG, triglycerides; Hcy, homocysteine; Glu, Glucose; Mon, Monocytes; Hb, hemoglobin;

ALP, alkaline phosphatase; GGT, γ-glutamyl transferase; CK-MB, creatine kinase isoenzyme;

α-HBDH, α-hydroxybutyrate dehydrogenase; BNP, Brain natriuretic peptide; cTnI, cardiac troponin I.

### Correlation between NK cell response to stimulatory factors and myocardial enzymes level in CAD patients

**Figure 3:**
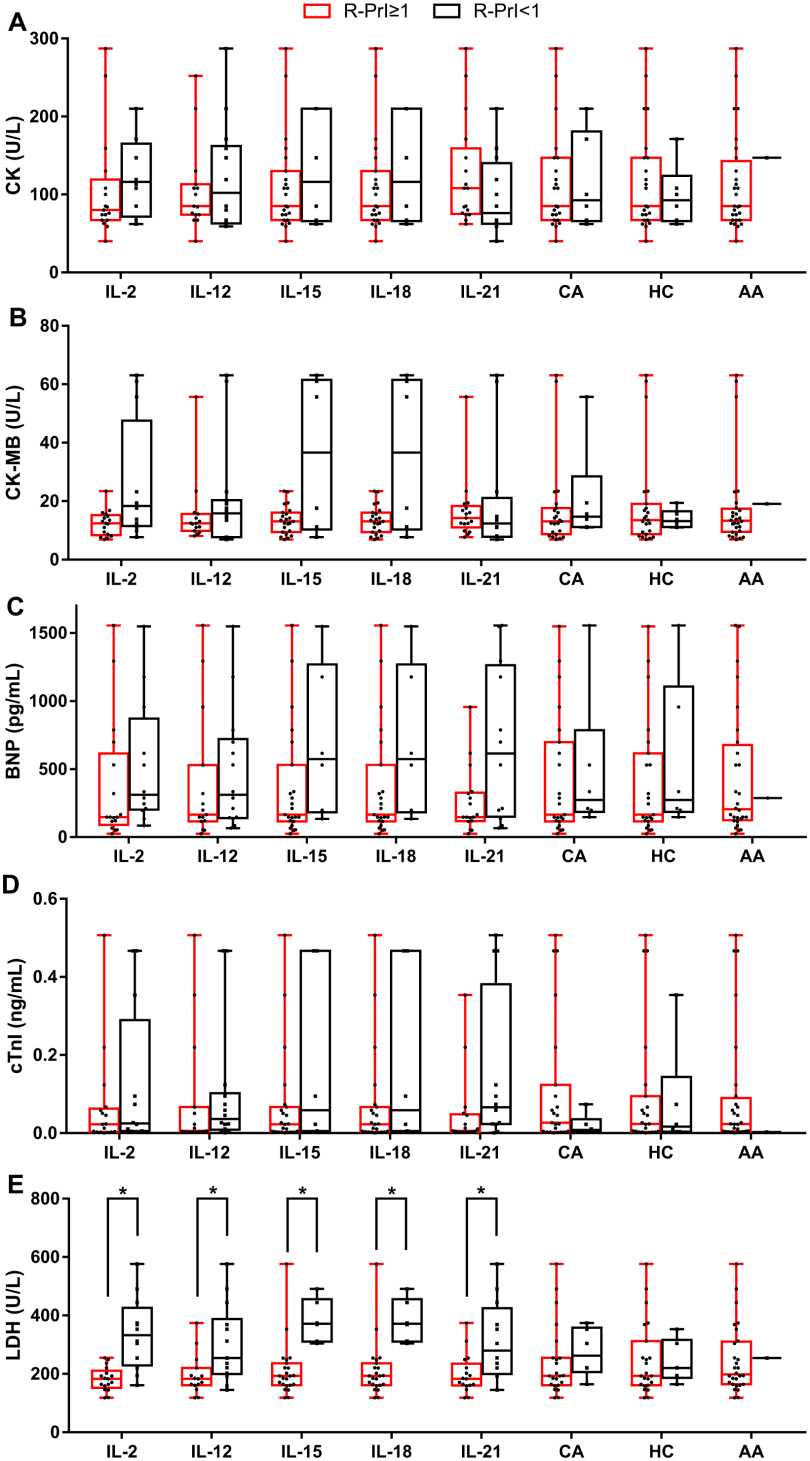
Correlation between the R-PrI of NK cells and serum myocardial enzyme level in CAD patients. Comparison of serum CK level **(A)**, serum creatine kinase isoenzyme (CK-MB) level **(B)**, serum BNP level **(C)**, serum cTnI level **(D)** and serum LDH level **(E)** between R-PrI >1 and R-PrI <1 NK cell group in responding to different stimulatory factors’ treatment. The normal range of CK is 20–173 U/L; the normal range of CK-MB is 0–25 U/L; the normal range of BNP is 15–450 pg/ml.; the normal range of cTnI is 0–0.02 ng/ml; the normal range of LDH is 114–240 U/L. Data were presented as mean ± SD; *P < 0.05.

**Figure 4:**
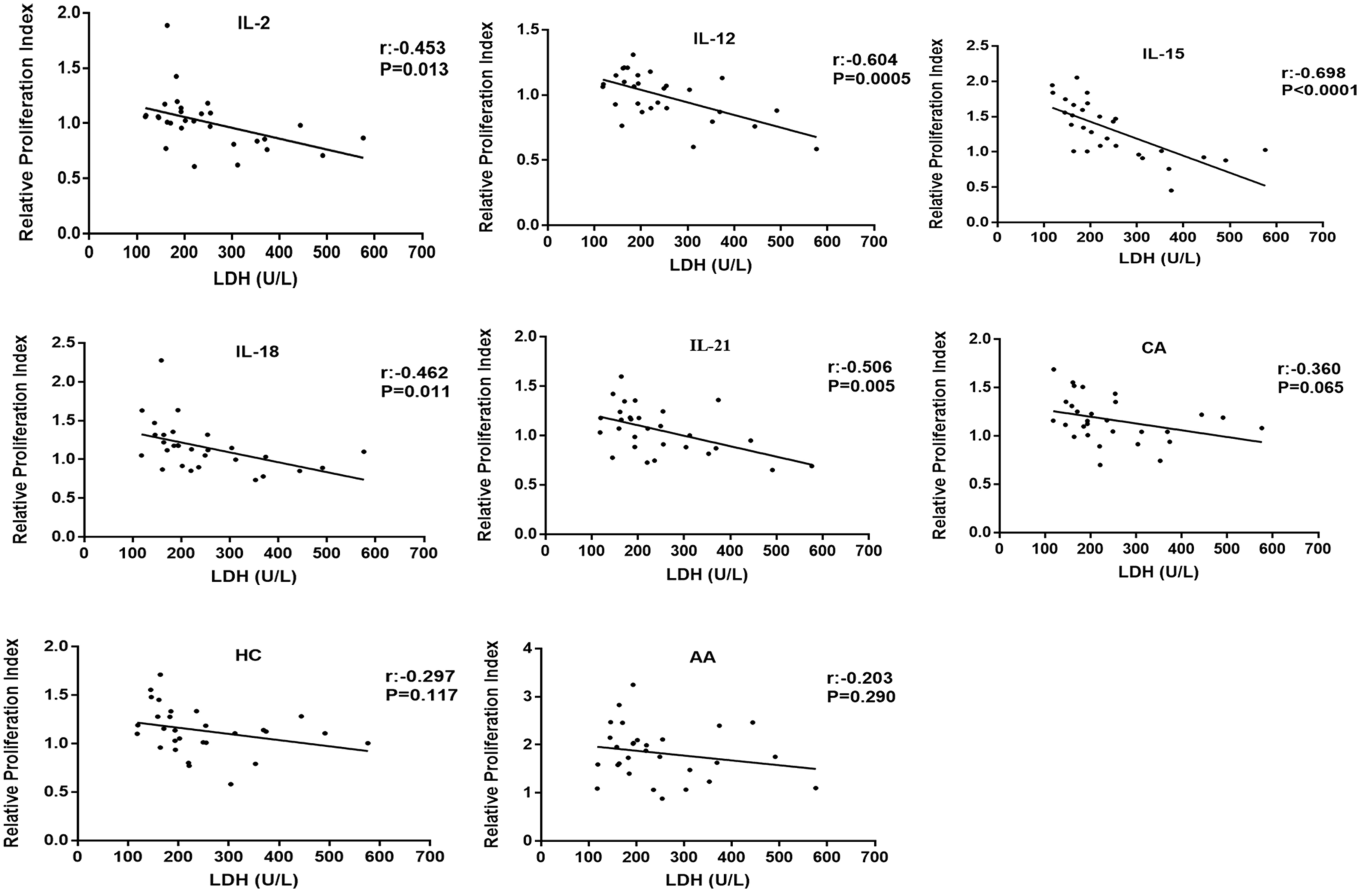
Correlation analysis of R-PrI of NK cells treated with eight stimulatory factors and serum LDH level in CAD patients. R-PrI of NK cells stimulated by each of the five interleukins is highly correlated with LDH levels. Spearman rank correlation analysis (r) and *P* values are provided in each graph.

Same stimulatory factors showed different effects on NK cell proliferative capability in different CAD patients; some were promotive while the others were inhibitory, indicating a response heterogeneity. Considering that the severity of coronary heart disease is closely related to myocardial enzyme level, we investigated the potential association of NK cell proliferative capability upon stimulation with the level of CK, CK-MB, BNP, cTnI, and LDH in the enrolled 29 CAD patients and 12 HDs (**Table 1**, **Supplementary Table S1**). The results showed that the proliferation of NK cells after CA or HC treatment was not related to the serum level of CK, CK-MB, BNP, cTnI, and LDH (**Figures 3**, **4**). When CAD patients’ NK cells were stimulated by IL-2, IL-12, IL-15, IL-18, or IL-21, the R-PrI >1 group and the R-PrI <1 group had no statistic difference in the serum CK level (**Figure 3A**), CK-MB level (**Figure 3B**), BNP level (**Figure 3C**) and cTnI level (**Figure 3D**). However, for LDH, no matter which interleukin was used, the LDH level of the R-PrI <1 group was significantly higher than that of the R-PrI >1 group in CAD patients (**Figure 3E**); serum LDH level was negatively correlated the proliferative capability of NK cells from CAD patients in responding to interleukins stimuli *in vitro* (**Figure 4**). In contrast, no correlation were observed between serum LDH level and the proliferative capability of NK cells from HDs (**Supplementary Figure S2**), which can be attributed to the low LDH level in HDs under physiological status. These results suggest that LDH may be an important indicator for judging whether NK cells in CAD patients have proliferative responses to interleukins *in vitro*.

### Correlation between circulating NK cell counts and serum LDH level in CAD patients

**Figure 5:**
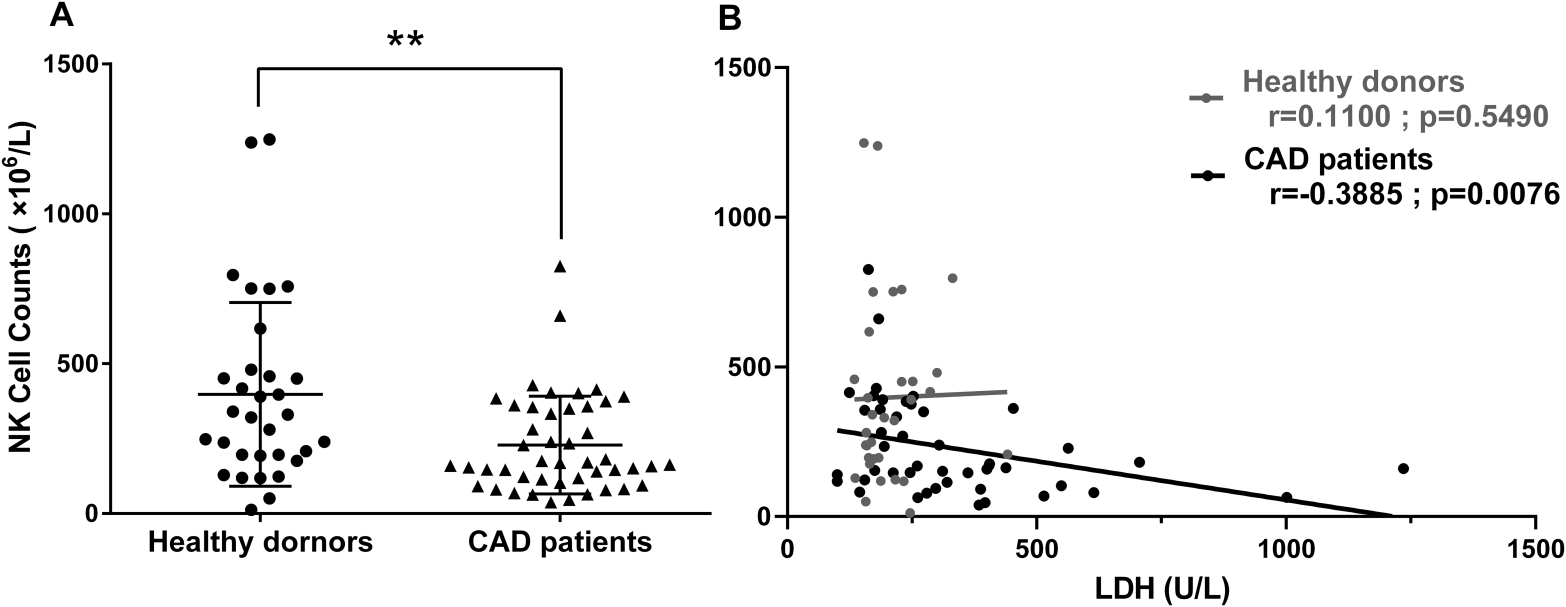
The relationship between circulating NK cell counts and serum LDH levels was estimated in CAD patients and healthy subjects. The flow cytometry data of peripheral blood lymphocyte counting from 46 CAD patients and 32 healthy donors were analyzed retrospectively. The absolute NK cell numbers **(A)** and its relationship with serum LDH level **(B)** were shown. Data were presented as mean ± SD; **P < 0.01. Spearman rank correlation analysis (r) and *P* values are provided in each graph.

The inverse correlation between serum LDH level and the *in vitro* proliferative capacity of NK cells in response to interleukins drove us to explore the potential relationship between the serum LDH level and the peripheral blood NK cell counts in CAD patients. Th*e* clinical data of 46 CAD patients and 32 healthy donors subjected to circulating TBNK cell counting were collected and retrospectively analyzed (**Table 2**). As shown in **Figure 5A**, the average NK cell counts in CAD patients was lower than that in healthy individuals. Noticeably, the serum LDH level was negatively correlated with the absolute NK cell counts evaluated in CAD patients but showed no relationship with that in healthy individuals (**Figure 5B**). In contrast, the serum LDH level was negatively correlated with the absolute counts of CD4^+^ and CD8^+^ T cells in both CAD patients and healthy individuals (**Supplementary Figures S3A, 3B**), but negatively correlated with the B cell number just in the healthy subjects (**Supplementary Figure S3C**). These results further suggested that the serum LDH level in CAD patients may function as indicator for peripheral blood NK cell counts.

**Table 2:** Characteristics of CAD patients and healthy donors subjected to NK cell counting.

	CAD patients	Healthy donors
Age (years)	69.39 (10.49)	60.03 (10.95)
Male/female (n)	27/19	18/14
Blood pressure		
Syst (mmHg)	133.21 (25.51)	123.77 (12.08)
Diast (mmHg)	79.59 (14.67)	76.05 (9.19)
Laboratory variables		
TC (mmol/L)	4.28 (1.41)	5.14 (1.21)
LDL-C (mmol/L)	2.28 (0.99)	2.66 (0.76)
HDL-C (mmol/L)	1.14 (0.44)	137 (0.48)
Apo-A1 (g/L)	0.96 (0.29)	1.19 (0.29)
Apo-B (g/L)	0.74 (0.25)	0.86 (0.20)
TG (mmol/L)	1.48 (0.70)	1.66 (0.85)
Hcy (µmol/L)	11.74 (3.03)	9.94 (1.58)
Glu (mmol/L)	6.57 (2.56)	5.68 (2.46)
Leukocyte subsets		
WBC (×10^9^/L)	9.72 (4.75)	7.06 (4.18)
NEUT (×10^9^/L)	69.03 (24.70)	64.94 (10.80)
LYMPH (×10^9^/L)	14.33 (11.11)	25.48 (9.99)
Mon (×10^9^/L)	0.63 (0.31)	0.47 (0.26)
RBC (×10 ^12^/L)	4.08 (1.05)	4.36 (0.94)
Hb (g/L)	116.94 (34.07)	128.47 (21.70)
Liver function indicators		
ALT (U/L)	29.15 (29.22)	22.9 (15.33)
AST (U/L)	41.82 (67.12)	21.47 (10.41)
ALP (U/L)	87.33 (82.34)	78.57 (24.58)
GGT (U/L)	60.83 (77.52)	36.17 (26.58)
Cardiac marker		
CK (U/L)	257.52 (583.846)	81.04 (64.49)
CK-MB (U/L)	35.80 (77.80)	17.5 (9.35)
LDH (U/L)	329.80 (221.51)	204.58 (65.59)
α-HBDH (U/L)	245.14 (145.02)	158.80 (52.90)
BNP (pg/ml)	959.33 (1176.78)	93.10 (70.08)
cTnI (ng/ml)	4.69 (11.42)	0.012 (0.022)

Values are given as mean (SD); abbreviations are described in **Table 1**.

### The effects of various stimuli on secretion of cytokines in NK cells

**Figure 6:**
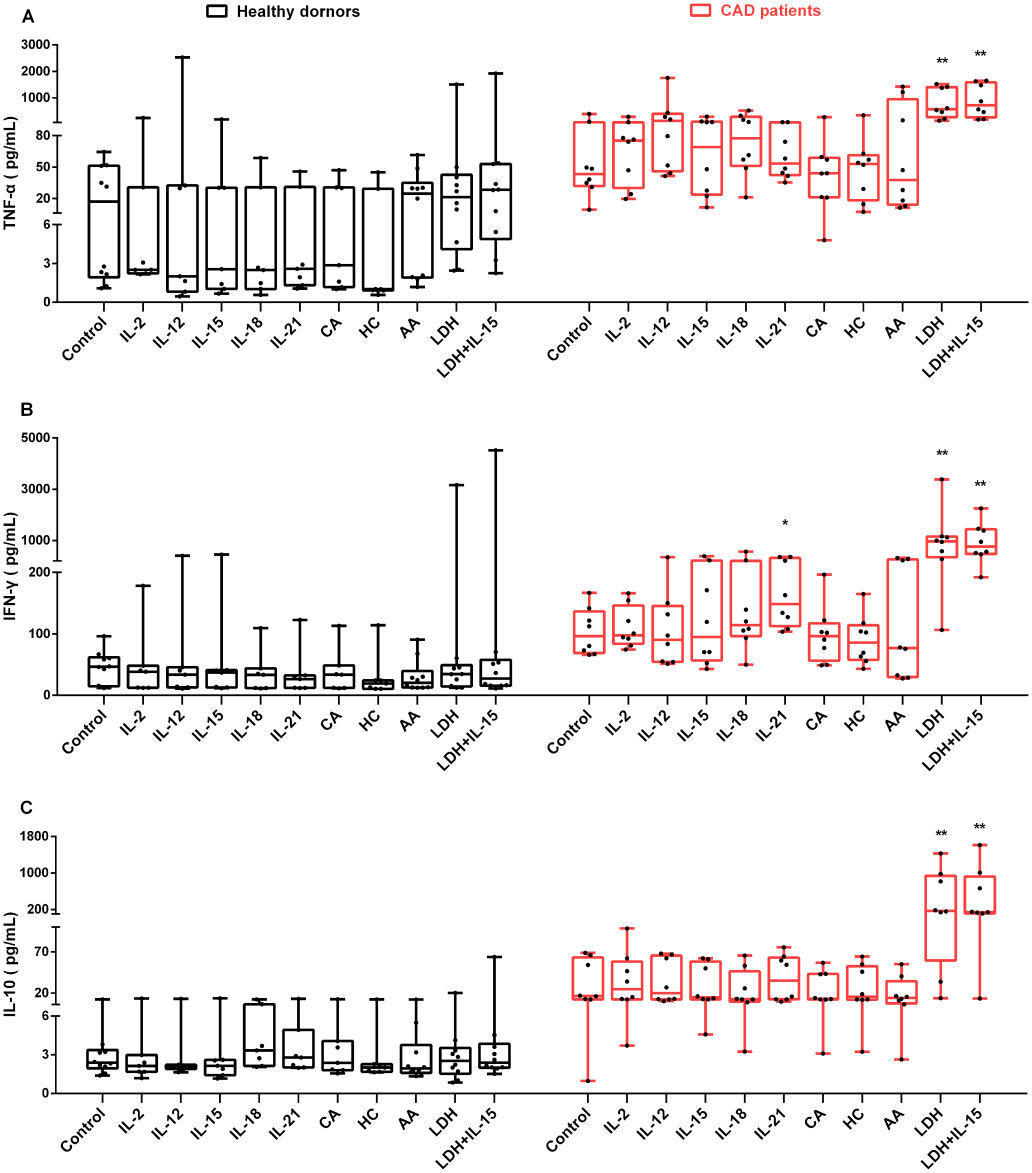
The influence of various stimuli on production of secreted IFN-γ, TNF-α and IL-10 in NK cells *in vitro*. NK cells were purified from peripheral blood of 10 healthy subjects and 8 CAD patients. After individual treatment with IL-2, IL-12, IL-15, IL-18, IL-21, CA (cortisone acetate), HC (hydrocortisone), and AA (ascorbic acid), lactate dehydrogenase (LDH), and LDH combination with IL-15 (LDH + IL-15) for 80 h, the supernatants of the cultured NK cells were collected, and the concentration of TNF-α **(A)**, IFN-γ **(B)**, and IL-10 **(C)** were measured. The untreated cells were used as the control. Data were presented as mean ± SD; *P < 0.05, **P < 0.01.

Given that NK cells could release various cytokines simultaneously upon stimuli, the secretion of IFN-γ, TNF-α and IL-10 were investigated in NK cells from HD and CAD patients. NK cells were purified from peripheral blood of eight CAD patients and ten healthy subjects (**Table 3**) and then detected the concentration of secreted IFN-γ, TNF-α and IL-10 after treatment with cytokines, glucocorticoids, AA and LDH for 80 h, untreated cells were used as the control. As shown in **Figure 6**, the presence of LDH alone or LDH plus IL-15 strongly enhanced the expression of IFN- γ, TNF-α and IL-10 in the NK cells from the CAD patients but not the NK cells from the healthy subjects; the presence of Il-15 alone did not show the promotion. These results suggested that LDH might play an important role in regulating immunologic responses in CAD patients; in contrast, the presence of IL-2, IL-12, IL-15, IL-18, IL-21, glucocorticoids as well as AA just showed weak promoting role or not at all. Altogether, NK cells could respond to LDH stimulation by changing the expression of IFN-γ, TNF-α or IL-10, and serum LDH at abnormally high level might be involved in CAD pathogenesis by regulating immunologic responses. However, this speculation needs further experimental confirmation due to limited sample numbers and heterogeneous responses.

**Table 3:** Characteristics of CAD patients and healthy donors enrolled for cytokines secretion by NK cells upon stimulation.

	CAD patients	Healthy donors
Age (years)	72.38 (9.4)	69.86(10.76)
Male/female (n)	4/4	7/3
Blood pressure		
Syst (mmHg)	120.5 (9.94)	128 (20.25)
Diast (mmHg)	73.38 (4.72)	79.17 (9.62)
Laboratory variables		
TC (mmol/L)	4.73 (1.44)	4.35 (0.78)
LDL-C (mmol/L)	2.92 (1.18)	2.38 (0.49)
HDL-C (mmol/L)	1.24 (0.25)	1.5 (0.35)
Apo-A1 (g/L)	1.10 (0.22)	1.29 (0.3)
Apo-B (g/L)	0.88 (0.30)	0.71 (0.15)
TG (mmol/L)	1.40 (0.60)	1.14 (0.45)
Hcy (µmol/L)	11.83 (1.68)	13.26 (3.41)
Glu (mmol/L)	6.06 (1.54)	5.08 (0.44)
Leukocyte subsets		
WBC (×10^9^/L)	9.03 (3.43)	5.96 (0.94)
NEUT (×10^9^/L)	6.43 (3.43)	3.48 (0.51)
LYMPH (×10^9^/L)	1.93 (1.07)	2 (0.52)
Mon (×10^9^/L)	0.54 (0.26)	0.45 (0.15)
RBC (×10 ^12^/L)	4.55 (0.3)	4.57 (0.45)
Hb (g/L)	132.25 (20.97)	145 (14.28)
Liver function indicators		
ALT (U/L)	21.13 (9.46)	22.43 (10.66)
AST (U/L)	20.13 (7.55)	23.71 (4.57)
ALP (U/L)	69 (23.71)	74.43 (20.16)
GGT (U/L)	35.5 (19.99)	22.43 (12.39)
Cardiac marker		
CK (U/L)	75.5 (32.92)	104.29 (45.2)
CK-MB (U/L)	18.23 (6.89)	16.17 (4.02)
LDH (U/L)	224.13 (39.7)	173.5 (41.36)
α-HBDH (U/L)	161.75 (27.29)	142.50 (36.98)
BNP (pg/ml)	261.36 (393.79)	29.28 (25.35)
cTnI (ng/ml)	0.025 (0.054)	0.0086 (0.0052)

Values are given as mean (SD); abbreviations are described in **Table 1**.

## Discussion

CAD is considered as a disease whose etiology might be at least partially related to chronic infections and a prolonged inflammatory state. A reduction of NK cells and a concomitant loss of NK cell function can be seen in CAD patients (7, 8). NK cells are classified into two major subsets according to their CD56 surface density. CD56^bright^ NK cells can produce immune-regulating cytokines, while the CD56^dim^ subset comprises more than 90% of NK cells and is associated with cytotoxicity (25). Persistent antigen-mediated activation of CD56^dim^ NK cells followed by apoptosis may cause the reduction of NK cells in CAD patients (26). Schiller et al. used atherosclerosis-susceptible mice to show that the atherosclerosis process is independent of NK cell-mediated cytotoxicity (27). However, Whitman et al. found that NK cells could promote the development of atherosclerotic lesions (28). It remains to be clarified whether the NK cell deficit is relevant to the atherosclerotic process, as well as the characteristics of NK cells in CAD patients. In this study, we investigated the *in vitro* effects of eight stimulatory factors, including IL-2, IL-12, IL-15, IL-18, IL-21, CA, HC, and AA, on the proliferation of NK cells and in CAD patients, and correlated the results with myocardial enzyme detection.

IL-2, IL-12, IL-15, IL-18, and IL-21 could promote NK cells entry into S and G2/M phases of the cell cycle in cancer patients or healthy subjects, and thus have been used to induce *ex vivo* NK cell proliferation (16, 29–33). These five interleukins function through activation of the Jak/STAT pathways in NK cells (34). Here we reported that IL-15 and IL-18 showed similar proliferative promotion in NK cells from CAD patients and healthy subjects, while the other three interleukins did not show statistically significant promotion (**Figures 1A, B**). The possible reasons may be explained by short treatment time and individual differences. Glucocorticoids were reported to increase the expression of activated NK cell receptors such as NKG2D and NKp46, enhance IFN-γ secretion, and increase the tumor-killing activity of NK cells (22). Our results showed that CA and HC could promote NK cell proliferation (**Figure 1**), however, neither CA nor HC had the capability of up-regulating the expression of IFN-γ, TNF-α, and IL-10 in NK cells (**Figures 6A–C**). The possible reasons could be attributed to a small size of sample number and individual difference.

AA serves as an antioxidant agent and contributes to immune defense by supporting various cellular functions in the innate and adaptive immune system (35). AA has also shown to promote the differentiation and proliferation of B cells and T cells (36, 37). While few reports on AA affecting NK cell proliferation, Huijskens et al. reported that addition of AA could promote NK cell proliferation and result in higher cell numbers without influencing NK cell functionality (38). Other studies showed that AA promoted a dose-dependent decrease in NK cell-mediated cytotoxicity (39, 40). Our results (**Figures 1A, B**, **2**, **6**) are consistent with the findings of Huijskens et al. (38). However, the underlying mechanisms remain elusive. In contrast, other tested stimulatory factors showed variable effect on NK cells proliferation. These results strongly suggest that different mechanisms may be used by AA, interleukins, and glucocorticoids. AA might affect NK cell proliferation more than NK cell function, while this speculation needs to be investigated further.

CK, CK-MB and LDH are important indicators of myocardial injury. When cardiomyocytes are damaged, they will increase rapidly (41). LDH is a tetramer enzyme of the glycolytic pathway, belonging to the 2- hydroxy acid oxidoreductase family. The role of this enzyme is to catalyze the reversible conversion of pyruvate to lactic acid and to oxidize nicotinamide adenine dinucleotide dehydrogenase (NADH) to NAD+ in the final step of the glycolysis pathway (42). LDH is associated with the severity of myocardial infarction. Furthermore, the more serious the heart failure, the higher the serum LDH levels (43, 44). The relationship between serum cardiac marker level and NK cell proliferation and function was not reported previously. Here we reported a negative correlation between serum LDH level and NK cell proliferative in response to the stimuli of tested interleukins in CAD patients (**Figures 3E**, **4**). Similar result was found in metastatic melanoma patients; those with elevated serum LDH level showed a low NK cell activity and NK cell receptor expression upon stimuli of IL-2, IL-12, and their combination (45). However, the underlying mechanisms are still unknown. Recently, Sheppard et al. (46) showed that LDHA-mediated aerobic glycolysis is necessary for NK cell activation by using a NK cell-specific genetic ablation of lactate dehydrogenase A (LDHA), which may provide some clues to explore the mechanisms behind negative correlation between NK Cells proliferation and serum LDH level.

Anti-inflammatory therapy has been proposed as a promising treatment for coronary heart disease (CHD) that could reduce residual inflammation risk (RIR) and therefore major adverse cardiovascular events (47). Inflammation plays a role in all stages of atherosclerosis, including accumulation of foam cells, formation of fatty streaks and fibrous plaques, rupture of acute plaques, and formation of thrombus, eventually leading to atherosclerosis and thrombotic complications (48). Previous studies have found that increased plasma TNF-α levels directly trigger foam cell formation and host NK cell release large amounts of IFN-γ to promote inflammatory processes, leading to plaque formation and instability (49, 50). Interestingly, the higher percentages of IL-10+ NK cells found in AMI patients, which also plays an important role in regulating NK cell function and healing damaged hearts (10). We observed that the presence of LDH strongly enhanced the expression of IFN-γ, IL-10 and TNF-α in NK cells *in vitro* comparing to the presence of IL-2, IL-12, IL-15, IL-18, or IL-21 (**Figures 6A-C**), suggesting a potentially important role of LDH in regulating immune responses. To the best of our knowledge, this is the first report of LDH influence on cytokines production in NK cells, suggesting that LDH may function as an immune-regulatory factor. Similar results were found by Sheppard et al. that LDHA plays an active role in antiviral and anti-tumor activities by promoting NK cell activation receptor signaling and effector functions (46).

Given that CAD patients usually have a reduction in peripheral blood NK cells and a concomitant loss of NK cell activity (51, 52), our results suggest that abnormally high level of serum LDH may be involved in CAD pathogenesis through regulating NK cell immune responses. However, due to small size of sample number, more samples and more experiments are needed to elucidate the effect of serum LDH level on NK cell activity and function *in vitro* and *in vivo*, including but not limited to NK cell proliferation, cytotoxicity, cytokines and receptors expression, and cell subsets.

## Data Availability

The raw data supporting the conclusions of this article will be made available by the authors, without undue reservation.
