# Supplementary material for: Proliferation capability of natural killer cells upon cytokines stimulation correlated negatively with serum lactate dehydrogenase level in coronary artery disease patients
Source: Front Immunol. 2024 Sep 2;15:1436747. doi: 10.3389/fimmu.2024.1436747 (PMC11402710; doi:10.3389/fimmu.2024.1436747)
Supplement: Supplementary file 1 [file DataSheet1.docx]

Supplementary Material

#
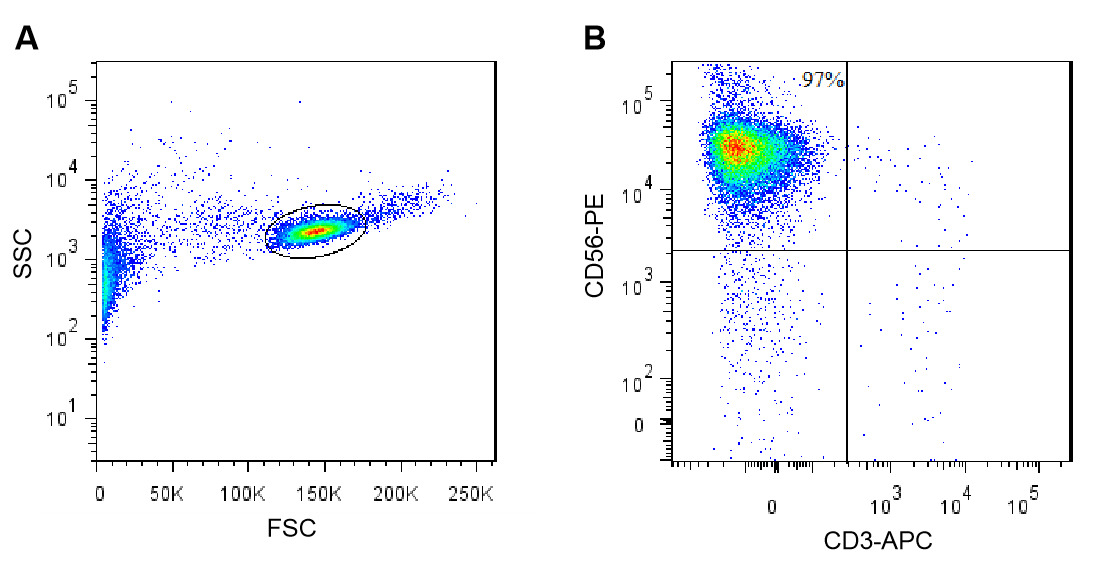


Figure S1. The purity of NK cells was detected by using flow cytometry. Primary human NK cells were isolated from PBMCs using the NK Cell Isolation Kit (Miltenyi Biotec, Bergisch Gladbach, Germany) according to the manufacturer’s instructions. After being fluorescently stained with CD56-PE and CD3-APC ((BD Biosciences, Franklin Lakes, USA), the cells were analyzed by flow cytometry. (A) Cell debris and dead cells Cell debris and dead cells were excluded from the analysis based on scatter signals. (B) NK cells were marked with CD56^+^ CD3^－^.

Table S1 The relative-proliferation index (R-PrI) of NK cells upon stimuli and serum myocardial enzyme level in the 29 CAD patients and 12 HDs

| Subject # | The R-PrI of NK cells in the Subjects treated with eight stimulatory factors | | | | | | | |  | Serum myocardial enzyme levels | | | | |
| --- | --- | --- | --- | --- | --- | --- | --- | --- | --- | --- | --- | --- | --- | --- |
|  | IL-2 | IL-12 | IL-15 | IL-18 | IL-21 | CA | HC | AA |  | CK (U/L) | CK-MB (U/L) | LDH (U/L) | BNP (pg/mL) | cTnI (ng/mL) |
| CAD-1 | 0.86 | 0.87 | 0.76 | 0.76 | 0.87 | 1.04 | 1.14 | 1.63 |  | 210 | 63.1 | 369 | 1176 | 0.467 |
| CAD-2 | 0.71 | 0.88 | 0.88 | 0.88 | 0.65 | 1.19 | 1.11 | 1.75 |  | 85 | 7.7 | 491 | 1643 | 0.452 |
| CAD-3 | 1.43 | 1.31 | 1.60 | 1.60 | 1.18 | 1.51 | 1.28 | 1.73 |  | 74 | 12.4 | 183 | 320 | 0.003 |
| CAD-4 | 1.01 | 1.10 | 1.01 | 1.01 | 1.16 | 0.99 | 0.96 | 1.61 |  | 85 | 15.6 | 164 | 147 | 0.004 |
| CAD-5 | 0.98 | 0.76 | 0.92 | 0.92 | 0.95 | 1.22 | 1.28 | 2.47 |  | 147 | 61.00 | 444 | 1548 | 0.095 |
| CAD-6 | 1.02 | 1.18 | 1.50 | 1.50 | 0.73 | 0.89 | 0.80 | 1.88 |  | 100 | 11.0 | 220 | 1555 | 0.023 |
| CAD-7 | 1.09 | 0.90 | 1.09 | 1.09 | 0.91 | 1.35 | 1.01 | 2.11 |  | 59 | 13.5 | 255 | 787 | 0.059 |
| CAD-8 | 0.61 | 0.90 | 1.09 | 1.09 | 1.07 | 0.70 | 0.77 | 1.99 |  | 171 | 19.4 | 221 | 333 | 0.011 |
| CAD-9 | 0.62 | 0.60 | 0.91 | 0.91 | 1.00 | 1.04 | 1.11 | 1.48 |  | 62 | 17.6 | 312 | 615.8 | 0.023 |
| CAD-10 | 0.77 | 1.21 | 1.52 | 1.52 | 1.24 | 1.55 | 1.45 | 1.58 |  | 113 | 9.8 | 161 | 245 | 0.005 |
| CAD-11 | 0.87 | 0.59 | 1.03 | 1.03 | 0.69 | 1.08 | 1.00 | 1.10 |  | 119 | 23.2 | 576 | 84.7 | 0.027 |
| CAD-12 | 1.09 | 0.94 | 1.19 | 1.19 | 0.75 | 1.16 | 1.33 | 1.06 |  | 159 | 7.3 | 236 | 65.3 | 0.001 |
| CAD-13 | 1.06 | 0.93 | 1.56 | 1.56 | 0.78 | 1.11 | 1.55 | 2.15 |  | 67 | 6.9 | 145 | 530.4 | 0.024 |
| CAD-14 | 1.03 | 0.87 | 1.28 | 1.28 | 1.18 | 1.23 | 1.05 | 2.10 |  | 287 | 16.8 | 202 | 147 | 0.002 |
| CAD-15 | 1.20 | 1.07 | 1.34 | 1.34 | 1.16 | 1.10 | 1.33 | 1.40 |  | 108 | 12.2 | 185 | 115 | 0.005 |
| CAD-16 | 0.84 | 0.80 | 1.01 | 1.01 | 0.82 | 0.74 | 0.79 | 1.24 |  | 62 | 13.8 | 353 | 211 | 0.074 |
| CAD-17 | 1.07 | 1.08 | 1.84 | 1.84 | 1.18 | 1.69 | 1.19 | 1.59 |  | 67 | 14.2 | 119 | 50.4 | 0.013 |
| CAD-18 | 0.96 | 1.09 | 1.69 | 1.69 | 1.35 | 1.01 | 0.94 | 2.04 |  | 108 | 12.5 | 194 | 955 | 0.354 |
| CAD-19 | 1.00 | 1.21 | 2.05 | 2.05 | 1.35 | 1.25 | 1.15 | 2.46 |  | 74 | 16.1 | 171 | 120 | 0.003 |
| CAD-20 | 0.76 | 1.13 | 0.45 | 0.45 | 1.36 | 0.94 | 1.12 | 2.40 |  | 210 | 55.7 | 374 | 529 | 0.006 |
| CAD-21 | 0.81 | 1.04 | 0.96 | 0.96 | 0.88 | 0.91 | 0.58 | 1.07 |  | 67 | 11.2 | 304 | 198 | 0.004 |
| CAD-22 | 1.05 | 1.15 | 1.75 | 1.75 | 1.42 | 1.35 | 1.48 | 2.47 |  | 76 | 8.7 | 146 | 147 | 0.22 |
| CAD-23 | 1.11 | 1.15 | 1.84 | 1.84 | 0.99 | 1.15 | 1.03 | 3.25 |  | 40 | 8.1 | 193 | 1292.6 | 0.507 |
| CAD-24 | 1.18 | 1.05 | 1.43 | 1.43 | 1.10 | 1.05 | 1.01 | 1.75 |  | 252 | 13.1 | 249 | 165.4 | 0.067 |
| CAD-25 | 1.89 | 1.21 | 1.67 | 1.67 | 1.60 | 1.52 | 1.71 | 2.83 |  | 130 | 23.4 | 164 | 53.4 | 0.051 |
| CAD-26 | 1.14 | 0.93 | 1.01 | 1.01 | 0.88 | 1.12 | 1.14 | 2.03 |  | 63 | 14.8 | 193 | 697.5 | 0.124 |
| CAD-27 | 1.17 | 0.76 | 1.39 | 1.39 | 1.07 | 1.31 | 1.28 | 1.95 |  | 80 | 7.7 | 159 | 142.2 | 0.046 |
| CAD-28 | 0.972 | 0.966 | 1.47 | 1.317 | 1.244 | 1.435 | 1.184 | 0.881 |  | 147 | 19.1 | 254 | 287.5 | 0.003 |
| CAD-29 | 1.105 | 1.063 | 1.944 | 1.051 | 1.031 | 1.158 | 1.101 | 2.859 |  | 84 | 9.4 | 118 | 25 | 0.003 |
| HD G1-1 | 0.800 | 0.876 | 0.765 | 0.785 | 0.656 | 1.228 | 0.961 | 1.737 |  | 57 | 12.7 | 114 | 19.2 | 0.001 |
| HD G1-2 | 1.011 | 1.188 | 1.509 | 1.391 | 0.964 | 0.995 | 1.287 | 1.993 |  | 117 | 21.2 | 229 | 10.0 | 0.005 |
| HD G1-3 | 0.857 | 0.886 | 1.01 | 0.897 | 0.879 | 1.195 | 1.148 | 1.888 |  | 104 | 8.7 | 203 | 11.1 | 0.008 |
| HD G1-4 | 1.028 | 1.105 | 1.559 | 1.225 | 0.953 | 1.353 | 1.687 | 2.401 |  | 70 | 13 | 221 | 9.8 | 0.005 |
| HD G1-5 | 1.328 | 1.051 | 1.672 | 1.146 | 0.883 | 1.257 | 1.566 | 2.293 |  | 152 | 14.3 | 147 | 35.0 | 0.001 |
| HD G1-6 | 1.167 | 0.997 | 2.056 | 1.258 | 1.119 | 1.199 | 1.378 | 2.042 |  | 164 | 24.5 | 241 | 20.4 | 0.014 |
| HD G2-1 | 0.615 | 0.815 | 0.706 | 0.567 | 0.861 | 0.641 | 0.594 | 1.698 |  | 60 | 16.2 | 184 | 29.3 | 0.008 |
| HD G2-2 | 0.724 | 1.310 | 1.123 | 0.861 | 0.729 | 0.919 | 0.810 | 1.589 |  | 88 | 17.3 | 233 | 36.0 | 0.012 |
| HD G3-3 | 1.252 | 0.946 | 1.010 | 0.933 | 1.049 | 0.760 | 0.682 | 1.853 |  | 53 | 12.4 | 179 | 15.3 | 0.014 |
| HD G4-4 | 0.753 | 0.728 | 0.680 | 0.808 | 0.577 | 0.980 | 0.941 | 1.806 |  | 178 | 11.4 | 156 | 10.3 | 0.001 |
| HD G5-5 | 0.882 | 1.035 | 0.800 | 1.343 | 1.219 | 1.321 | 0.816 | 2.516 |  | 97 | 22.6 | 206 | 13.9 | 0.010 |
| HD G6-6 | 0.629 | 2.042 | 0.802 | 1.287 | 1.291 | 1.476 | 1.032 | 2.185 |  | 148 | 17.3 | 148 | 70.9 | 0.006 |

CAD, Coronary artery disease; HD G1，Healthy donors Group 1; HD G2，Healthy donors Group 2;


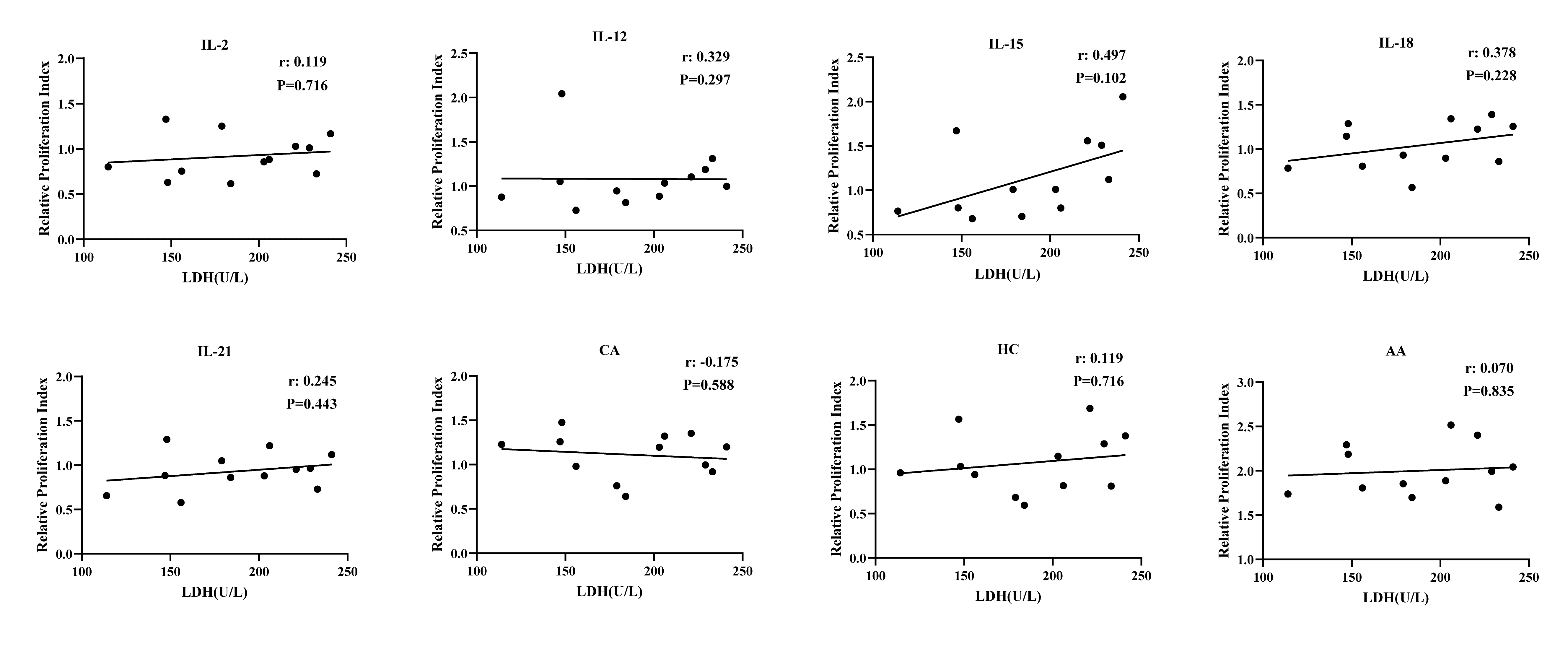


**Figure S2.** Correlation analysis of R-PrI of NK cells treated with eight stimulatory factors and serum LDH level in 12 healthy donors. R-PrI of NK cells stimulated by each of the eight stimulatory factors are not correlated with LDH levels. Spearman rank correlation analysis (r) and *P* values are provided in each graph.

**

**

Figure S3. Relationship between circulating lymphocyte cell counts and serum LDH levels was estimated in CAD patients and healthy subjects. The clinical data of 32 CAD patients and 46 healthy individuals subjected to peripheral blood lymphocytes analysis with flow cytometry were analyzed retrospectively. The absolute numbers of CD4+ T cells, CD8+ T cells, and B cells, as well as their relationships with serum LDH levels, were shown on left and right panel of (A), (B), and (C), respectively.
